# Anterior segment optical coherence tomography for the detection of silicone oil emulsification on the iris surface

**DOI:** 10.1038/s41433-024-03261-4

**Published:** 2024-07-29

**Authors:** Mona Karsten, Matteo Morello, Imke Lau, Vasyl Druchkiv, Ines Valente Lopes, Simon Dulz, Christos Skevas, Martin Stephan Spitzer, Luca Mautone

**Affiliations:** 1 Department of Ophthalmology, University Medical Center Hamburg-Eppendorf, Hamburg, Germany; 2 Cardiology School, University of Brescia, Brescia, Italy

**Keywords:** Tomography, Diagnosis

## Abstract

**Background:**

To investigate the presence of silicone oil (SO)-emulsification on the anterior iris surface with anterior segment optical coherence tomography (AS-OCT).

**Methods:**

In this single-center cross-sectional study, vitrectomized eyes with SO tamponade that underwent AS-OCT imaging and gonioscopy examination during the postoperative follow-up visits, were reviewed.

**Results:**

45 eyes of 42 consecutive patients were included. In 35.6% of the eyes (*n* = 16) emulsified SO droplets were detected in the anterior chamber (AC) angle by gonioscopy and in 55.6% (*n* = 25) on the anterior iris surface by AS-OCT imaging. The presence of SO emulsifications in the AC-angle correlated with the presence of SO emulsifications on the anterior iris surface (OR = 13.4, 95% CI [2.179–82.130]; *p* = 0.005). The accuracy of the AS-OCT predicting the presence of SO in the AC-angle was 71.0% and the sensitivity was 87.5%. No significant association between SO droplets in the AC and other clinical parameters including endotamponade-duration or type of silicone oil were found. The presence of emulsified SO droplets on the anterior iris surface detected by AS-OCT was significantly correlated to postoperative IOP rise (*p* = 0.027).

**Conclusion:**

AS-OCT is a suitable method for the detection of SO on the anterior iris surface. SO droplets on the iris surface correlate with elevated postoperative IOP and with the presence of SO in the AC detected by gonioscopy, therefore AS-OCT might be used as a screening method for the detection of SO migration into the AC.

**Trial registration number:**

Institutional Review Board of the Hamburg Medical Chamber (Ethik-Kommission der Ärztekammer Hamburg): 2023-300372-WF.

## Introduction

Since its introduction in 1962 by Cibis et al, silicone oil endotamponade (SOT) has been frequently used in retinal surgery [1, 2], showing good anatomical and functional results in eyes with retinal detachment complicated by severe proliferative diabetic retinopathy, proliferative vitreoretinopathy, trauma or chronic uveitis with marked hypotony [2–4].

Besides its advantages of long-term retinal tamponade effect, suppression of anterior segment neovascularization in diabetic patients [3–5], early visual rehabilitation [3], and its long-term safety record [2], there are also several disadvantages. Complications provoked by silicone oil (SO) can affect both the posterior and the anterior segment [2]. In particular, postoperative intraocular pressure (IOP) rise related to oil emulsification and oil migration into the anterior chamber (AC) has been frequently described in the literature [6–8]; The incidence varies from 8% up to 67.4% [7, 9–11].

Several techniques for the visualization of SO are already in use: SO in the AC can be identified with slit-lamp biomicroscopy and the presence of emulsified SO in the AC-angle can be detected by gonioscopy [12, 13]. Studies conducted with ultrasound biomicroscopy (UBM) demonstrated this examination as a reliable instrument for the visualization of SO in the AC [7, 14–20]. However, just as gonioscopy, UBM represents a method that is associated with patients’ discomfort.

The primary aim of our study was to investigate whether anterior segment optical coherence tomography (AS-OCT) is a suitable non-invasive and time-efficient method for the detection of SO emulsification on the iris surface. In addition, we analysed possible risk factors for the presence of emulsified SO on the iris and in the AC-angle.

## Materials and methods

The study adhered to the tenets of the Declaration of Helsinki. Approval of the Institutional Review Board (Ethic Committee of the Medical Chamber Hamburg, procedure number: 2023-300372-WF) was obtained.

In this retrospective single-center cross-sectional study, patients were identified from the clinical database (IFA, IFA Systems AG, Frechen, Germany) between January 01, 2022, and August 01, 2023, at the Ophthalmology Department of the University Medical Center Hamburg-Eppendorf, Germany. The inclusion criteria were: (1) previous pars plana vitrectomy (PPV) with silicone oil tamponade (SOT) for retinal detachment, (2) postoperative anterior segment OCT (AS-OCT) and (3) gonioscopy before SOT removal, both examinations performed on the same day at least 30 days after PPV. Eyes with ectopia lentis, zonular weakness, history of trauma, history of previous intraocular surgeries other than cataract extraction or vitrectomy for retinal detachment, dislocated IOL, or aphakia were excluded.

Electronic medical records of patients were reviewed for the collection of ophthalmic history, visual acuity, intraocular pressure (IOP), use of IOP-lowering eyedrops, type of SOT (2000 centistoke [cs] or 5000cs), previous PPV, presence of pseudophakia at the time of surgery, combined surgery with cataract extraction, endotamponade duration, anterior segment biomicroscopic slit-lamp examination, and dilated fundal examinations. Postoperative IOP rise was defined as newly detected IOP ≥ 21 mmHg and/or introduction of IOP-lowering eyedrops due to postoperative IOP elevation. The presence of emulsified SO droplets in the anterior chamber (AC) angle was examined by gonioscopy (3-Mirror Lens, Volk, Optical, Mentor, USA). To investigate the presence of emulsified SO droplets on the iris surface, swept-source AS-OCT imaging (SS-OCT, Topcon, Tokyo, Japan) was performed using either a radial scan (6.0 mm; *N* = 8; 17,8%), a line scan (3.0 × 3.0 mm; *N* = 3; 6,6%), or a single line scan (3.0 mm; *N* = 34; 75,6%) focusing on the upper half of the iris. Regardless of the method chosen, 3 scans were performed: at 9, 12 and 3 o’clock. Based on Madanagopalan’s et al case report, AS-OCT is performed as part of our routine practice if oil emulsification in the anterior chamber is suspected in the slit lamp examination as an attempt to capture it in multimodal imaging [21]. Based on several previously published studies, emulsified SO droplets mainly seem to appear as small, hyperreflective, and roundish structures in OCT examination [19, 21–24]. In this study the abnormalities on the iris surface presented themselves as small, mostly roundish structures in varying reflectivity in AS-OCT examination - comparable to those identified by Grewal et al and Madanagopalan et al who also studied oil emulsifications in the anterior chamber [21, 23]. It therefore can be assumed that they are also oil emulsifications. The presence of the iso- and hyperreflective structures in AS-OCT was independently evaluated by three investigators (MK, IL and LM).

Fisher’s Exact Test and multivariate binomial logistic regression analysis were performed with R version 4.1.2. The significance level was set at *p* < 0.05. Test quality criteria (e.g. sensitivity, specificity, accuracy—see Table 2) were determined using a confusion matrix.

## Results

In this study, 45 eyes of 42 consecutive patients were included. The median BCVA amounted to 0.9 LogMAR (range 1.7–0.2). At the time of PPV, 62.2% (*n* = 28) of the eyes were already pseudophakic while 31.1% (*n* = 14) underwent combined cataract extraction. The mean endotamponade duration was 106.3 days (range 37–1499 days). In 60% of the eyes (*n* = 27) 2000cs SOT was used and in the other eyes (40%) 5000cs SOT. 62.2% of the eyes (*n* = 28) were PPV naïve and 26.7% (*n* = 12) underwent a repeat PPV with SOT.

**Fig. 1:**
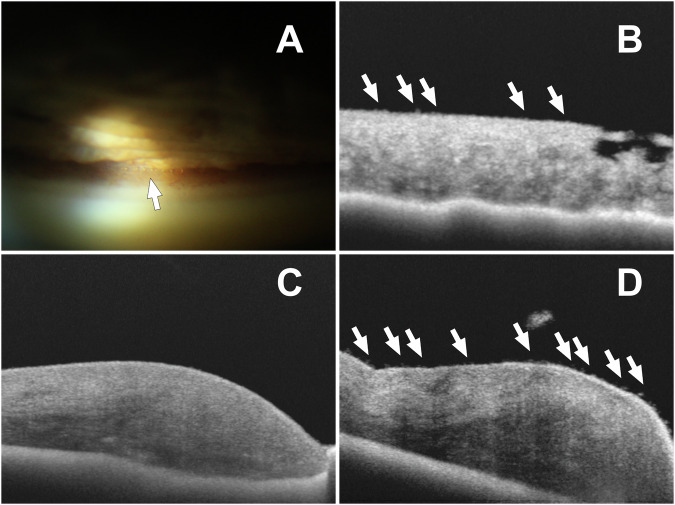
Silicone oil droplets in the anterior chamber. **A** Silicone oil (SO) droplets in the chamber angle detected by gonioscopy. **B** SO droplets on the anterior iris surface detected by swept-source AS-OCT. **C** AS-OCT shows normal findings of the iris surface of the non-operated eye. **D** Silicone oil (SO) droplets in the contralateral eye after vitrectomy with SO.

In 35.6% of the eyes (*n* = 16) emulsified SO droplets were detected in the AC-angle by gonioscopy (see Fig. 1A). In all the cases, SO droplets were located in the superior half of the AC-angle only and covered 3 clock hours of the AC-angle (Median [Q1; Q3]: 3.0 [1.75; 3.0] range 1–4). No hyperoleon was described.

The presence of clinically visible SO droplets on the iris surface was not noted in the clinical examination by slit lamp biomicroscopy. AS-OCT imaging detected emulsified SO droplets on the iris anterior surface in 55.6% (*n* = 25) of eyes (see Fig. 1B, D).

The accuracy of AS-OCT for predicting visible SO on gonioscopy was 71% [95% CI (55.7%, 83.6%)]. Thereunder 31% were true positives (TP) and 40% were true negatives (TN). The adjusted accuracy (Kappa measure) was 44%, which indicates a moderate agreement. The sensitivity (TP/TP + FN) was 87.5% and the specificity (TN/TN + FP) was 62.1%. The error rate was 29% and the disagreement was significant according to McNemmar’s Test (*p* = 0.027). For the confusion matrix on which the calculations are based, see Table 1.

**Table 1:** Confusion matrix.

		SO on iris surface (AS-OCT)	
		No	Yes
SO in the anterior chamber angle (Gonioscopy)	No	18	11
	Yes	2	14

*SO* silicone oil, *AS-OCT* anterior segment optical coherence tomography.

The relationship between the presence of SO emulsifications in the AC-angle and on the anterior iris surface was also confirmed in the multivariate binomial regression analysis (OR = 13.376, 95% CI [2.179–82.130]; *p* = 0.005). Running a backward selection algorithm based on Akaike Information Criteria (AIC) on the full model retained only SO on the iris surface as a predictive variable. No other significant association between SO droplets in the AC-angle and other clinical parameters were found—investigated variables are shown in Table 2.

**Table 2:** Multivariate binomial logistic regression analysis.

	SO in the anterior chamber angle (OR [95% CL]; *P* value)
Age (years)	0.958 [0.881–1.041]; 0.314
Sex	0.379 [0.049–1.577]; 0.149
Cataract surgery	1.912 [0.137–26.717]; 0.630
Pseudophakia	2.188 [0.187–25.639]; 0.533
Oil type	1.000 [0.999–1.001]; 0.799
Previous PPV (n)	0.723 [0.183–2.851]; 0.643
Previous PPV with SO	2.459 [0.215–28.188]; 0.470
Endotamponade duration (days)	0.997 [0.987–1.007]; 0.541
SO on iris surface (AS-OCT)	13.376 [2.179–82.130]; 0.005

*SO* silicone oil, *SO type* 5000 or 2000 centistoke [cs], *PPV* pars plana vitrectomy (PPV), *AS-OCT* anterior segment optical coherence tomography.

Concerning the analysis of the intraocular pressure in one case no intraocular pressure measurement was noted, consequently, this eye was excluded from the examination. An IOP equal to or above 21 mmHg was measured in 15.9% (*n* = 7) of the eyes and a topical IOP-lowering therapy was introduced after PPV in 11.4% (*n* = 5). The presence of SO emulsifications in the AC-angle was not significantly correlated to postoperative IOP rise—see Table 3. In contrast, the presence of emulsified SO droplets on the anterior iris surface was significantly correlated to postoperative IOP rise (*p* = 0.027).

**Table 3:** Association between the presence of silicone oil (SO) in the anterior segment and the presence of SO on the iris surface detected by anterior segment OCT with the intraocular pressure (IOP).

Gonioscopy				
	No presence of SO in anterior chamber angle (*n* = 28)	Presence of SO in anterior chamber angle (*n* = 16)	Total (*n* = 44)	*P* value
IOP rise	4 (14.3%)	6 (37.5%)	10 (22.7%)	= 0.133

AS-OCT				
	No presence of SO on iris surface (*n* = 19)	Presence of SO on iris surface (*n* = 25)	Total (*n* = 44)	*P* value
IOP rise	1 (5.3%)	9 (36.0%)	10 (22.7%)	= 0.027

IOP rise: eyes with postoperative IOP ≥21 and/or post PPV introduction of IOP lowering therapy. Fisher’s Exact Test.

## Discussion

Migration of SO into the AC after vitrectomy with SOT is a well-known postoperative phenomenon [7, 8, 21, 25]. Several methods for the detection of SO have already been described including UBM, electron microscope, coulter counter, and OCT [7, 14–17, 19, 20, 26].

In this study, AS-OCT was investigated as a method for SO detection on the iris surface. In 55.6% of the eyes, small reflective SO-droplets on the anterior iris surface were made visible by using swept-source AS-OCT. To the best of our knowledge, this is the first study that systematically evaluated AS-OCT as a tool for the detection of SO droplets on the iris surface in eyes with SOT. Up to now, the use of AS-OCT for imaging of SO in the AC was only documented in 3 case reports: Mishra et al. and Grewal et al. described retrocorneal SO detection by OCT [17, 23], whereas Madanagopalan et al. reported SO detection in the canal of Schlemm [21]. Concerning the detection of SO on the iris surface, UBM was used as a diagnostic method. Grigera et al. examined 34 eyes to establish reproducible UBM imaging patterns of SO in the AC, detecting SO on the iris surface in 64.7% of the eyes [16]. Interestingly, the detection rate was higher than in the current study (55.7%), even though the patients were examined in the supine position, and oil is known to migrate upwards due to its lower density than aqueous humor [23]. A possible reason for the higher detection rate could be the relatively high rate of included eyes with aphakia (64.8%)—a known risk factor for oil migration [2, 27]. Another reason could be that in the current study mainly single line scans were performed (75,6%) - since the complete iris surface was not visualized, a relevant false negative rate cannot be ruled out. In principle, UBM seems to be a sensitive method for oil detection: In a large study by Zhao et al. with 113 patients, oil in the AC was detected by UBM in 100% of cases [26]. An advantage of using UBM is the possibility of both quantifying volumes and finding oil in various locations – not only in the AC. SO was detected also behind the posterior surface of the iris when UBM was employed [14]. However, UBM is time-consuming, may cause patients’ discomfort, and is not readily available everywhere.

A noteworthy disadvantage of UBM and as well of the SO-detection by AS-OCT is that very small oil emulsifications probably cannot be made visible. Chan et al. used a coulter counter multisizer to find out that over 65% of the emulsified SO droplets in their samples had a diameter smaller than 2 μm and thus not detectable by light microscopy [20]. Therefore, AS-OCT—with a resolution not higher than 8 µm—is also not a suitable method for optically displaying such small oil emulsifications [28]. The detection of SO emulsifications likely will remain a diagnostic challenge since Wickham et al. were able to show oil emulsification even in the optic nerve itself by histopathological detection [29].

In the current study, oil droplets were detected in 35.6% of the eyes using gonioscopy, a result within the range described in the literature (22.2–64%) [6, 30]. The presence of SO in the AC-angle detected by gonioscopy was also compared with the presence of SO emulsification on the iris surface identified by AS-OCT. Taking gonioscopy, as an already established method and using AS-OCT to predict it, an accuracy of 71% was shown [95% CI (55.7%, 83.6%)]. Thereunder 31% were true positives and 40% were true negatives. The adjusted accuracy (Kappa measure) was 44%, which indicates a moderate agreement. Interestingly the false positive rate was relatively high (24%), especially in comparison to the relatively low false negative rate (4%) which leads to the assumption that AS-OCT is a more sensitive method for SO detection in the AC. This is also reflected in the calculated sensitivity of 87%. An important point to consider for this analysis is that the occurrence of SO was not only compared by two different diagnostic methods but also at two different locations, so deviations were to be expected. To test whether there was still sufficient agreement between the two methods, a multivariate binomial logistic regression analysis was used revealing a significant correlation between the presence of emulsified oil droplets in the anterior chamber angle and on the iris surface (*p* < 0.005).

These findings are encouraging for using AS-OCT as a more sensitive screening method for the detection of SO emulsification in the anterior chamber. Furthermore, it offers the advantages of being non-invasive, time-saving, and reproducible.

In our study, the presence of SO in the AC did not correlate with the number of previous vitrectomies or with the previous use of SOT. Despite the higher viscosity, stability, and inferior emulsification tendency of 5000ct compared to 2000ct, the SO type did not influence the SO migration into the anterior segment [2, 31]. The SOT duration had no effect as well. This seems comprehensible due to the relatively short duration of the SOT in this study (mean: 106.31 days; median: 56 days) since generally, a period of 2–6 months following the primary surgery is advocated for SOT removal [10, 23].

Besides the detection of SO on the iris surface and checking on potential risk factors for SO migration, we also studied the relation between postoperative IOP and SO in the AC as the postoperative IOP elevation is a well-known problem of SOT [6, 7, 10, 26, 32]. In the current study, 37,5% of eyes with SO in the AC-angle and 36,0% of eyes with SO on the iris surface had an IOP rise. According to the literature, the incidences vary noticeably with a rate up to 67,4% [10]. The presence of SO in the AC-angle via gonioscopy did not correlate with a postoperative rise—defined eyes as postoperative IOP ≥ 21 and/or postoperative introduction of IOP lowering therapy—whereas the detection of SO by AS-OCT on the anterior iris surface was significantly associated with postoperative IOP rise (*p* = 0.027). On the one hand, this difference could be due to the higher SO-detection rate by using AS-OCT (35.6% vs 55.6%). Avitabile et al. showed a strong correlation between the incidence of high intraocular pressure and the quantity of emulsified SO in the AC measured by UBM [7]. Even though we didn’t quantify the SO findings, the higher detection rate could indicate an increased occurrence of SO in the AS. On the other hand, this difference could be due to the detection of SO droplets in a limited section of the AC-angle only (3.0 clock hours) – as a large portion of the AC-angle remains open for the outflow of aqueous humor.

SO droplets on the iris surface detected by AS-OCT might reflect a dispersion of microbubbles throughout the AC and the whole outflow system. The higher IOP in eyes with SO on the iris surface might be due to the limited resorption of aqueous humor via the uveal system. Wickham et al. who studied nine enucleated globes that had previously undergone retinal detachment surgery with SO could show ultrastructural evidence of silicone in the trabecular meshwork and immunohistochemically a macrophage response associated with oil in the meshwork [29]. They suggested that the trabecular infiltration by silicone oil and the associated trabeculitis may be a reason for the raised intraocular pressure [29].

The current study is limited by its retrospective, monocentric study design, the restricted number of cases, and the lack of possible quantification of SO droplets using AS-OCT. Especially regarding the latter point, a comparative analysis of UBM and AS-OCT would be desirable. Furthermore it can be assumed that the false negative rate with regard to oil detection in AS-OCT is relatively high, as a majority of patients have only received a single line scan which only covers a fraction of the iris surface. It also should be noted that no definite proof could be provided that the observed structures are oil emulsifications as this would only be achievable by surgical anterior chamber lavage with subsequent analysis of the sample using a coulter counter multisizer. However, our assumption is based on the observations of previously published studies in which emulsified SO droplets mainly seem to appear as small, hyperreflective, and roundish structures in OCT examination [19, 21–24]. In this study, in which oil emulsifications on the iris surface are to be visualized for the first time using AS-OCT, the abnormalities on the iris surface presented themselves only slightly different as small, mostly roundish structures with either iso- or hyperreflectivity. The impression of a different reflectivity could arise from the fact that the iris surface, in whose comparison the droplets were placed, itself exhibits its own tissue-specific reflectivity.

It would be desirable if the results of this study could be compared in a standardized form with the results of other studies. Only recently, the so-called ITEMS score was published by Romano et al., which intends to serve as a standardized recording of intraocular oil emulsifications [12]. The score consists of the examination results from slit lamp microscopy, gonioscopy, and fundus examination under mydriasis and OCT [12]. It considers not only the qualitative but also the quantitative detection of intraocular oil emulsifications [12]. However, clinical validation of the score is still outstanding at present. Nonetheless, it seems to be a promising tool in the future for the comparability of study results in the context of intraocular emulsion of silicone oil [12].

In conclusion, AS-OCT is a suitable method for the detection of SO on the anterior iris surface in eyes that underwent vitrectomy with SOT. SO droplets on the iris surface correlate with elevated postoperative IOP and with the presence of SO in the anterior chamber detected by gonioscopy. Therefore AS-OCT might be used as a screening method for the detection of SO migration into the AC. Since more than half of the eyes (55.6%) present SO on the iris surface, prospective randomized trials are advoked to clarify the role of anterior chamber irrigation during SOT removal.

## Summary

### What was known before

- Migration of silicone oil into the anterior chamber is a known problem after pars plana vitrectomy with silicone oil endotamponade and can lead to an increase in IOP.
- For oil detection in the anterior chamber, gonioscopy or UBM, both time-consuming and uncomfortable techniques for the patient, have been mostly used so far.

### What this study adds

- AS-OCT is a suitable method for the detection of silicone oil on the anterior iris surface.
- Silicone oil droplets on the iris surface correlate with elevated postoperative IOP and with the presence of silicone oil in the anterior chamber detected by gonioscopy.
- Therefore AS-OCT as a non-invasive time-saving and reproducible diagnostic method might be used as screening method for the detection of silicone oil migration into the anterior chamber.

## Data Availability

The datasets generated during and/or analysed during the current study are available from the corresponding author on reasonable request.
